# Experiences of a remote, person-centred intervention in older persons living with frailty - a qualitative study

**DOI:** 10.1186/s12877-025-06509-0

**Published:** 2025-10-15

**Authors:** Zahra Ebrahimi, Patricia Olaya-Contreras, Mahboubeh Goudarzi, Inger Ekman

**Affiliations:** 1 Institute of Health and Care Sciences, Sahlgrenska Academy, University of Gothenburg, Centre for Person-Centred Care, University of Gothenburg (GPCC), Gothenburg, Sweden; 2 Department of Medicine, Geriatrics and Emergency Medicine, Sahlgrenska University , Gothenburg, Sweden

**Keywords:** Person-centred care, Person-centered care, Frailty, Older people, EHealth, Remote care, Qualitative study

## Abstract

**Background:**

Globally, healthcare systems are challenged with implementing effective home-based self-management programmes for older persons living with frailty within a proactive, integrated model of remote, person-centred care (PCC).

**Aim:**

This study explores the experiences of a remote PCC intervention in older persons living with frailty.

**Methods:**

A qualitative inductive conventional content analysis was conducted using semi-structured telephone interviews. The intervention involved older adults who received PCC through a combined approach of telephone calls and a digital platform. Fourteen **c**ommunity-dwelling older people (≥ 75 years, screened as frail) with recent emergency care visits but had not been hospitalised, were included.

**Results:**

Participants felt secure due to the engaging, empathetic and professional-friendly nature of the telephone conversations. In contrast, many informants reported difficulty using the digital platform due to its complexity.

**Conclusion:**

Telephone-based, person-centred remote care enhanced resource availability and promoted feelings of security among older persons living with frailty. However, further development of user-friendly digital tools for older people is still needed.

**Trial registration of the main project:**

ClinicalTrial.gov: NCT04416815. Registered 07/06/2021.

**Supplementary Information:**

The online version contains supplementary material available at 10.1186/s12877-025-06509-0.

## Background

Old age is often characterised by multimorbidity, frailty and excessive sensitivity to negative stressors, including illnesses [1]. Frailty is usually a common ageing process affecting all organ systems, leading to reduced reserve capacity and increased risk of dependence when performing daily activities [2]. Older persons living with frailty necessitate comprehensive, holistic, interprofessional health care interventions integrating multiple social and health care sectors [1, 2].

A recent review emphasised the importance of co-designing integrated, person-centred care (PCC) by involving older people and their informal caregivers in collaborative processes [3]. PCC facilitates collaboration, ensuring parity between the patient’s experiential understanding of illness and the health professional’s clinical expertise [4–7]. The foundation of PCC practice is the collaborative relationship between patients and staff. This is made mainly by listening to the patient’s illness stories of how it affects daily life, particularly reinforcing the capabilities and resources revealed in the narratives, jointly agreeing on a personal health plan and securing the partnership by documenting the process and agreement in the patient record, which should be available to both partners [5–9].

Studies indicate that robust and secure eHealth solutions may significantly aid in providing PCC for older adults residing at home [10–12].

Digital health care devices have positively affected older people (e.g., increased daily activities, accessibility to care and reduced hospitalisations) [13–15]. While eHealth shows promise in enhancing care coordination and management for older people with multi-morbidity, major implementation challenges persist due to funding mechanisms, structural barriers, and a lack of adequate technical support [10, 16]. Greenhalgh’s NASSS framework (Non-adoption, Abandonment, Scale-up, Spread and Sustainability) outlines seven critical domains that influence implementation and evaluation of health care technologies: the patient condition, the technology itself, the value proposition, the adopter system including staff, patients, caregivers, organisational factors, the wider healthcare system and interactions over time. The framework underlines that technology often fails not due to single isolated factors but because of complex interactions across these multiple domains [17].

Older people participating in a remote PCC intervention reported that they experienced increased access and involvement in care [18], suggesting that welfare technology may play a role in implementing PCC, but its adoption among older people remains limited [19]. MacInnes’s (2021) integrative review demonstrated that even the most advanced technologies often fail to achieve satisfactory levels of acceptability, accessibility and utilisation at the micro level [20]. Nonetheless, a substantial research gap persists concerning the experiences of older persons using remote PCC. This study aimed to explore the experiences of a remote PCC intervention in older persons living with frailty.

## Methods

The study employs a qualitative methodology grounded in knowledge co-creation and recognising the subjective nature of lived experiences and the interpretive role of researchers. This approach reflects an ontological stance of multiple realities, and an epistemological understanding of knowledge as collaboratively constructed [21, 22], thereby aligning with the constructivist principles of person-centeredness approaches [23, 24]. Semi-structured interviews were conducted using an interview guide [25] developed, discussed and refined by the present research group, which included patient representatives (see supplemental material). Qualitative inductive conventional content analysis [21] was employed to systematically organise and interpret the data into structured categories without theoretical preconceptions [22]. This approach is in line with qualitative research employing an inductive methodology, prioritising participants’ perspectives while using the theoretical framework as an interpretive lens rather than a rigid analytical structure [21, 22]. The authors followed the Standards for Reporting Qualitative Research reporting guidelines [22, 26].

### Patient and public involvement

The IHOPe project (Initiating Health Planning with and for Older People through e-Health) was designed in close collaboration with an advisory group comprising researchers, clinicians, patient representatives and health care professionals (HCPs) (including nurses, physiotherapists and physicians). An advisory group reviewed the platform and interview guide refining the clarity of the questions for participants. To further assess usability—including content, structure, and platform access—a patient representative (an 82-year-old woman with moderate frailty) who was familiar with digital tools and had collaborated with the research team throughout the project, tested both the guide and the platform prior to implementation. Her feedback was fully incorporated into the final versions.

### Setting

The IHOPe project was designed as a randomised controlled trial in an urban context in Western Sweden [27]. In addition to usual care, the participants in the intervention group received person-centred telephone support and the opportunity to communicate with an HCP through the digital platform for a 6 month period [27].

### The theoretical framework in the IHOPe project

Person-centeredness [23, 24] and the Capability Approach [28] are two congruent theoretical frameworks that guided the planning, development and implementation of the IHOPe project. Practicing PCC according to Ricœur’s ethic (“aiming at the ‘good life’ with and for others, in just institutions”) (p.172) [24] is an action ethic [5, 8] that guided our practical actions and goals in the intervention, i.e., acknowledging the patient as a person, engaging that person as an active partner in planning of their health care by listening to narrations of experiences of daily life. PCC means identifying health barriers and needs and confirming a patient’s capabilities and resources. The capabilities approach encompasses capabilities and the potential for functional achievement, contingent upon available resources and circumstances. In this context, health is linked to the capacity of older persons to pursue their objectives and activities while surmounting daily challenges. The development of PCC, facilitated by health promotion and preventive measures, seeks to ensure equitable access to optimal health outcomes, regardless of frailty [28, 29].

### Intervention components

The intervention components have been described elsewhere and were developed by a research group and a reference group encompassing patient representatives [27]. The intervention aimed to initiate partnership-building, encourage older people to describe their health situation and needs and further identify and engage their capabilities and resources. An initial telephone conversation prompted the intervention, with a follow-up appointment scheduled to facilitate intermittent contact with the older person via phone and digital platform. Participation in the platform, however, was not obligatory. Participants’ narratives of their everyday situation and their needs, resources and health-related goals were the core components of the telephone conversations. The length of the conversations varied. An HCP (a specialised registered nurse, a physiotherapist or an occupational therapist) started the conversation by recapitulating the aim of person-centred support, asking open-ended questions, listening to participants’ experience of their condition, context, needs and resources and co-creating achievable and relevant health-related goals. The following Person-centred conversations were often shorter and focused on reviewing goals, sometimes formulating new goals and discussing strategies to attain the goals.

The goals were documented in a health plan by the participant or the HCP and uploaded to the digital platform. In agreement with the older persons, the documented health plan was sent home by letter to those who did not use the digital platform. Contingent upon the agreements reached during the phone call, family, friends and additional health professionals would be invited to join the digital platform. In addition, the platform facilitated daily diary entries and self-monitoring using a 1–5 scale to track symptoms (e.g., pain, fatigue, anxiety) and general well-being (e.g., how well they had slept). Participants and the HCPs could also communicate through messages in a chat-like forum. Moreover, the platform contained links to other relevant web pages for participants to seek information or connections. Participants were advised to contact the national emergency healthcare services if a serious event occurred. Table 1 illustrates the key components of the intervention and its delivery.

**Table 1:** The components of the IHOPe remote Person-centred intervention [27]

Component	Description	Delivery
Person-centred phone calls	- Conducted by health care professionals trained in PCC. - First call within 2 weeks of inclusion; follow-ups scheduled in agreement with the participant. - Focus on narrative-based discussions to identify needs and resources and co-create health goals.	Mandatory for all participants. Scheduled contact and the participants spontaneously initiated contact occurred only during the daytime.
Health plan	- Co-created during calls and updated regularly. - Includes personalised goals, strategies and support needs.	If using a digital platform: Uploaded to the platform for shared access. If not using a digital platform: Sent via postal mail to ensure accessibility.
Digital platform (optional)	- Enables messaging, self-monitoring (e.g., symptom tracking) and access to health resources. - Shared with invited caregivers/professionals if participants agree.	Voluntary; only for participants who opted in. The digital platform was available continuously (24/7) during the first six months of the intervention.
Team collaboration	- Participants could invite family, friends or health care professionals to join the platform. - Facilitated shared decision-making	Linked to digital platform use (optional).
Health care professional training	- Workshops on PCC, ethics and remote person-centred dialogue.	Ensured fidelity to the intervention

### Sampling and participants

The participants, community-dwelling older people seeking emergency care but not hospitalised, were invited from the intervention group (*n* = 113) in the IHOPe trial [27]. They were ≥ 75 years old and screened as frail using the FRESH (Frail Elderly Support Research Group) instrument developed by the Frail Elderly Support Research Group [30], and cognitively functional (oriented to time, place and person). Because the FRESH screening instrument does not assess frailty severity levels, we employed the Clinical Frailty Scale (CFS) to obtain a more detailed gradation of participants frailty. Individuals in the terminal stages of life requiring palliative care were excluded, as they lacked a registered address, were enrolled in a competing randomised controlled trial, or exhibited cognitive impairment (disorientation to time, place and person) [27]. Interpretation services were offered to participants whose native language was not Swedish. Older persons with visual or hearing impairments were included in the study. Through strategic, purposive sampling, 14 older people living with frailty and diverse functional ability (seven men and seven women) were selected. The objective was to select participants with an even sex distribution, diverse frailty levels [31] and varying degrees of digital platform usage. A purposive sampling method was employed to collect comprehensive and varied perspectives, with the sample size determined based on pragmatic considerations such as time and resource efficiency, as well as the principle of information power to ensure rich and meaningful data relevant to the research questions [32]. All invited participants consented to participate, resulting in complete retention and no attrition.

### Data collection

Data were collected through 14 individual semi-structured telephone interviews with community-dwelling older persons, using a structured interview guide. Interviews with participants were held at their respective homes with open-ended questions and follow-up probes to explore older persons’ experiences with a remote, person-centred intervention. The introductory question was,

“*Would you like to tell me your thoughts and expectations after reading the information sheet about the remote person-centred care intervention?”*

Further questions explored participants’ circumstances and interactions with the intervention, encompassing telephone and digital communication, while avoiding predetermined answers. A specific example was requested:

*“Please describe a concrete situation*,* event or moment where this approach proved advantageous or challenging”.*

Through unrestrained conversation, participants were encouraged to describe their experiences with the intervention and its components. The interviewer facilitated open-ended discussion, allowing participants to articulate their perspectives on the intervention and its constituent elements. This inductive approach centred on participant accounts, prioritising their views over preconceived structures. All interviews were audio-recorded and transcribed verbatim. All data were anonymised at the point of transcription.

### Research team and reflexivity

Three female researchers, each possessing substantial professional health care experience and a history of geriatric care, conducted the interviews. The first interviewer was a specialist nurse working as a research coordinator for the IHOPe study; the second interviewer was an audiologist working as a research assistant; and the third interviewer was an occupational therapist holding a PhD and a researcher at IHOPe. All interviewers received training in qualitative methodologies and possessed extensive experience conducting in-depth interviews. The research coordinator initiated contact via telephone to recruit participants, providing details of the study’s purpose and addressing ethical and logistical aspects. No established relationships existed between the interviewers and participants. Grounded in person-centredness [23, 24] and the Capability Approach [28], the interview methodology specifically prioritised participant narratives. This was achieved through carefully designed open-ended questions thatemphasised an exploratory approach. The interviewers maintained a consciously curious stance, using follow-up questions to elicit deeper reflections while avoiding leading prompts. The theory was applied as an interpretive lens rather than a constrictive framework. This inductive approach ensured the findings remained grounded in participants’ experiences while maintaining theoretical relevance to person-centred care delivery and guided by person-centred care and the Capability Approach. Three additional researchers conducted the data analysis independently, further mitigating interpretive bias. This layered approach strengthened the trustworthiness of the findings by incorporating multiple perspectives and methodologies [22].

### Analysis

A qualitative inductive conventional content analysis was performed, involving iterative readings of each interview (vertical reading) and reading the whole text (horizontal reading) to identify emergent subcategories, categories and main categories reflecting informants’ perspectives on [21] the person-centred, remote intervention. The analysis involved five steps:

1. Two authors (ZE, POC) independently and repeatedly reviewed verbatim transcripts of all interviews to gain a comprehensive understanding of participants’ perspectives on the intervention.
2. The interview transcripts were imported into NVivo (version 1.7.1) software for line-by-line analysis, facilitating the construction of “meaning units”, words, sentences or paragraphs containing aspects related to each other in their content and context.
3. All meaning units from the 14 transcribed interviews with internal and external homogeneity were condensed and sorted into subcategories.
4. Subcategories containing comparable content were sorted into distinct categories.
5. The main category emerged through constant repetitive reading, interpretation and validation of the interviews in their entirety, condensed meaning units and comparisons of the subcategories and categories [33].

The NVivo software facilitated the data management process throughout the study. Two researchers (POC, ZE) independently conducted at least two analyses of all transcripts to ensure coding reliability. Any disagreements in transcription and coding were resolved through consensus discussion or consultation with the fourth author (IE). Table 2 illustrates the iterative progression of interpretive analysis and driving results from meaning units to the main category.

**Table 2:** The iterative progression of interpretive analysis and driving results from meaning units to the main category

Meanings unite	Condensed meanings unite	Sub-Categories	Categories	Main Category
Be listened to with empathy and interest. Feeling good talking with others who have an interest in me. An opportunity to amend one’s mindset, narrate and review one’s health issues and resources To identify feasible health goals and actions A telephone call is time beneficial. It is accessible when needed.	Be encouraged to narrate. Feeling good to talk A telephone call is a time-beneficial tool. An accessible tool and approach when needed.	Being encouraged to narrate Talk to someone who is listening with interest and empathy. Appropriate advice, encouragement and goal setting	Professional-friendly conversation with narration, confirmation, encouragement and goal setting	Fostering a sense of security through person-centred conversations and being in a continued relationship with health care professionals

### Ethical approval and informed consent

The study was conducted according to the principles of the Declaration of Helsinki and approved by the Regional Ethical Review Board in Gothenburg, Sweden (Dnr 2019–05364, Dnr 2020–03550, Dnr 2021–03255). We were guided by Kvale’s [25] suggestions for the ethical conduct of interview studies in study design and implementation. Informants received information about the purpose and procedure of the interview in writing and orally by telephone on the day of the interview. Participants received confirmation that participation was voluntary, allowing withdrawal from the study and interview at any time without compromising their care. The interview materials were confidential; therefore, any disseminated information would not permit the identification of individuals.

## Results

Fourteen older persons living with frailty participated in this study. All participants were Swedish speaking. Table 3 provides a more detailed description of participant characteristics.

**Table 3:** Participant characteristics (*n* = 14)

Age years	
Mean	80
Median (range)	79 (75–86)
Sex	
Female	7
Male	7
Frailty based on the Clinical Frailty Scale (CFS, range 0–9)	
Median (range)	4 (4–5)
Civil status	
Living alone	9
Married/partner	5
Education level	
Compulsory	2
Secondary school	3
University/High school	9
Telephone calls	
Number of telephone calls, median and (range)	4 (2–5)
Total length of telephone calls in minutes, mean/median and (range)	86.5/79.5 (54–165)
Number of persons using activities on the digital platform	
Accessing links in the digital platform	10
Writing messages to the HCP in the research group	10
Rating current health status	6
Confirming that informants read their health plan	11
Inviting team members (family and professionals)	1

Audio-recorded data were collected for 626 min (in average 45 min per participant). Table 4 presents an overview of the main category, categories and subcategories, followed by a detailed description.

**Table 4:** Overview of the results sorted into the main category, categories and subcategories

Main Category	Fostering a sense of security through person-centred conversations and being in a continued relationship with health care professionals		
Categories	Professional-friendly conversation with narration, confirmation, encouragement and goal setting	Being aware of one’s resources and building a sense of security	Communication via phone was favoured, whereas accessibility and usability of the digital platform were challenging
Subcategories	Being encouraged to narrate Talk to someone who is listening with interest and empathy Appropriate advice, encouragement and goal setting	Developing personal awareness and resourcefulness Communication access fostered a sense of security	The phone calls were beneficial, accessible and meaningful Struggling with signing in and working on the digital platform

### Professional-friendly conversation with narration, confirmation, encouragement and goal setting

The telephone conversations were valued for their amicable and professional nature. The conversations focused on participants’ daily challenges and the coping strategies they use. All participants experienced receiving good advice and helpful tips when needed. They also felt encouraged and affirmed in their ability to use resources and strategies to navigate their daily lives. Throughout the conversation, participants and the HCP undertook collaborative goal setting and the development of actionable strategies to facilitate participant goal attainment.

#### Being encouraged to narrate

Participants valued the opportunity and encouragement to articulate their health concerns, problems, resources and coping strategies. Participants reported positive emotional experiences during their interactions with HCPs, frequently using terms such as “feel good” or “it was nice.” The HCP facilitated writing participant narratives on the platform, with participants confiding feelings that were inexpressible to their networks (e.g., friends and relatives).

The conversations

*“…helped me to think and let go”*,* [I-18] “…get it [the narrations] out*,* helping me to reflect and let go” [I-46]. “I received concrete advice and a bit of help and could relieve myself of some troublesome things [I-56].”*

#### Talk to someone listening with interest and empathy

Participants described receiving empathetic and attentive audiences for their narratives. It felt like *“having someone by my side.”* Respondents believed that their complaints received due consideration from the HCP.

*“The conversations have been the best thing for me in this study because I felt I could talk to someone who listened*,* was empathic and showed interest.” [I-339]*.

Participants appreciated these professionally oriented conversations due to perceived time constraints among their standard care providers. Meaningful discussion and comprehensive understanding of their health issues and circumstances necessitated adequate time for dialogue with a compassionate and attentive individual, particularly when receiving or lacking pertinent diagnostic information.

#### Appropriate advice, encouragement and goal setting

Through a supportive and trusting professional relationship, participants received effective guidance on goal setting and the development of health-related strategies. Following acute illness or hospitalisation, the HCP provided pertinent counsel and information tailored to the participant’s needs. In addition, communication with the HCP was both timely and substantive.

*“You have a contact you can talk to. You can get good advice and the like. It is valuable.” [I-46]*.

*“The advice (from the health care staff) was on how to tackle and deal with problems as well as encouragement to go on.” [I-30]. “It was probably these pieces of advice (from the staff) on how to tackle and handle the problems one had*,* and a bit of encouragement to keep working on it and … You never encounter that at the health center” [I-18]*.

Managing the conversation, providing relevant information based on participant needs, and collaboratively establishing achievable health objectives were valued. Some participants could evaluate their planned goals, such as commencing an exercise regimen.

*“I think it is very good that the goals about your health and illness are clarified*,* and how to deal with them in the future.” [I-312]*.

### Being aware of one’s resources and building a sense of security

Participants experience enhanced feelings of security, attributed to increased awareness of personal and external resources facilitated by the phone dialogues. They also felt confident managing daily routines by prioritising essential aspects and strategically pacing their health objectives.

#### Developing personal awareness and resourcefulness

Participants reported that conversations with the HCP improved their health literacy and access to coping resources.

*“The conversations over the phone helped shape my thinking”. [I-44]*

*“The nurses in the project recommended that I ask personnel in health care questions about my treatment and test results*,* and I also received a little help with medical terms*,* so the conversations have given me a lot.” [I-339]*.

*“I was simply encouraged*,* and the feeling that I wasn’t managing well was removed.” [I-112]*.

The conversation and co-creation of a health plan’s content and structure were perceived as useful, prompting the participants to re-evaluate their resources and plans.

*“It is good that the patient him/herself makes a plan on what to do and how to contact health care”. “I think that making goals and strategies to manage my health and treatment was quite good; it helped to clarify how to handle it in daily life” [I-312].*

*“It’s very good to write if you feel tired or unwell*,* or whatever it is”. [I- 46]*

#### Communication access fostered a sense of security

Participants prioritised maintaining contact with health care professionals. The availability of professional assistance engendered feelings of security.

*“I think it’s absolutely excellent*,* the accessibility*,* ease of use*,* and feeling that there is always someone who quickly answers. That part was great.” [I-339]*.

*“That one can get in touch with health care; it is not just random contact with new people all the time. I think that is significant that the patient can reach health care smoothly and easily” [I-312].*

Participants found the platform’s supplementary health-related resources pertinent, such as links addressing insomnia, nutrition, wellness and other health topics. This intervention resulted in two participants establishing personal Facebook profiles and expanding their social networks.

### Communication via phone was favoured, whereas accessibility and usability of the digital platform were challenging

Participants uniformly valued telephone conversations for being simple and sufficient. However, many participants experienced difficulties logging into the digital platform, and some encountered further difficulties upon entry.

#### The phone calls were beneficial, accessible and meaningful

Participants valued the time-saving aspect of the remote health approach. Most participants favoured telephone communication as the optimal and most convenient method. However, two participants found telephone communication less personal than face-to-face interaction.

*“The conversations were extremely important for me.” [I-339]*

*“It would have been much better if we met face-to-face… or eye-to-eye. It would have made a bigger impression and stuck better than a phone call.” [I-41].*

#### Struggling with signing in and working on the digital platform

Participants encountered difficulties accessing the digital platform due to a complex, multi-step authentication process. In addition, the sign-in instructions were confusing and difficult to retain.

*“Many steps [were necessary], and sometimes it was difficult to log onto the platform.” [I-41]*.

Four participants declined to participate in the health plan’s development. Within this context, the platform presented usability challenges for participants with various impairments, including visual impairments, limited fine motor skills and physical limitations, such as pain. In contrast, two participants with prior computer experience reported a positive experience, describing the platform as an “*appealing challenge*.” However, these participants suggested the platform’s interactivity could be enhanced by implementing more pertinent queries, thereby mitigating the need for extensive written narratives and goal descriptions.

*“I’m quite computer-savvy*,* so I have no difficulty navigating those contexts. But I can imagine that a person my age would have certain difficulties… Yes*,* so many things one must remember.” [I-305]*.

*“I have some difficulty communicating on the computer.” [I-109].*

One participant, however, found diary writing on the platform to be a valuable experience, while others perceived it as a challenge.

*“Writing about my daily health situation and feelings in the diary was very nice.” [I-46].*

*“It has been difficult to express myself in words.” [I-339]*.*“It wasn’t that tempting because I had difficulty knowing what to write*,* and then I thought I was repeating myself.” [I-112]*.

*“It didn’t interest me.” [I-109*,* I-41]*.

*“I have not used digital interaction opportunities because I thought they do not offer additional benefits over traditional conversations.” [I-339].*

## Discussion

This study demonstrates that person-centred communication and readily available health care professionals significantly enhance feelings of security among older persons living with frailty. A remote, person-centred approach fosters collaborative partnerships between older persons and HCPs, enhancing awareness of individual resources and capabilities and promoting sense of security. These findings show the practical application of the intervention’s theoretical framework and corroborate previous results demonstrating the positive effects of PCC in older persons [34–36] and those with chronic diseases [37]. Systematic reviews indicate that person-centred interventions for older persons living with frailty in non-hospital settings are beneficial [38, 39]. The present results corroborate Ekstedt et al.’s user-friendly design, encouraging patient narrative expression, collaborative goal setting and documentation of personal goals [40]. Another person-centred study found that integrating older patients into their health care plans is an integral life-long process, not merely a singular event, promoting security and collaborative planning with the HCPs [36]. This study, along with previous research, demonstrates that partnership in a collaborative approach result in increased empowerment and self-understanding among both patients and HCPs [36, 40]. The application of person-centred principles [23, 24] and the Capability Approach [28] through collaborative goal-setting, empowered participants to reassess their circumstances and daily lives by identifying and validating their resources and coping strategies. This process, characterised by shared responsibility, capability building and equitable partnership, aligns with Social Cognitive Theory’s core tenets: the learning environment, personal engagement and socio-interactive development [41].

Our findings are supported by numerous studies that identify key factors contributing to effective narrative elicitation. These factors include establishing trusting patient-provider relationships, actively listening to patient accounts of their illness narrative, and collaboratively formulating personally meaningful care goals [41, 42]. In this respect, evidence supports the essential role of patients’ narratives in practising PCC [4]. Each HCP interacting with participants received training in person-centred philosophy, ethics and practice. Thus, theoretical and practical knowledge are required to establish a collaborative and respectful partnership. In a separate remote PCC intervention study, participants reported phone calls with HCPs as the most meaningful activity [42]. These findings corroborate our results, indicating that participants recognised the value of phone calls. Patients and staff described nine observable indicators of practising PCC in an interview study [43]. These key indicators include having a welcoming, interested and courteous reception, agreeing on the structure and aims of the conversation and eliciting patients’ wishes for the involvement of significant others [43]. The results of our study are consistent with these indicators, despite its remote execution.

The results presented here also align with a previous study showing the complex nature of remote, person-centred communication between HCPs and patients, where the patient’s priorities become paramount [44]. Previous research on remote health support demonstrated its contribution to patients´ self-management of chronic conditions, confirming our findings [40, 45]. Our participants preferred phone calls over the digital platform because of its simplicity and time-saving aspects. Still, the topics covered were thoroughly reviewed, and ongoing communication ensured participant reassurance. While existing research indicates a preference for face-to-face contact among patients [46–48], our findings suggest that phone and digital communication were particularly valued by participants during the COVID-19 pandemic’s social restrictions. Moreover, remote approaches enhance accessibility for individuals with mobility limitations while optimising efficiency by eliminating travel [48]. Our findings also correspond with those of Barenfeld et al. [18], who showed that participants valued the availability of HCPs for communication and advice. A core element of PCC practice involves supportive and empathetic professional-friendly interactions promoting trust, which, within the present study, engendered confidence and sense of security among older persons living with frailty.

The implementation and use of eHealth technologies for older people present a further ethical consideration: the tension between security and user-friendliness [48]. Participants found the platform login process to be complex. Physical impairments as well as a lack of digital knowledge or engagement, also constrained digital communication with HCPs. Our findings indicate positive experiences among older persons using telephone-delivered PCC interventions; however, the substantial variability in their acceptance of digital health solutions must be acknowledged. Although digital platforms enjoy considerable engagement from older persons other individuals face significant adoption barriers related to accessibility, technological literacy and personal preferences [46–48]. Previous studies have shown that the design of digital support systems for older persons living with frailty necessitates a person-centred approach that accounts for individual physical limitations, needs and capabilities [49–51]. Our findings further suggest the necessity of developing suitable digital tools tailored for older people living with frailty, achieved through their active participation [20, 52]. This study highlights two critical considerations for health care practice: First, while diverse digital communication tools are currently employed within healthcare systems, their sustainability and clinical efficacy require incorporating a PCC framework and training for health care professionals in remote PCC. Second, the choice of digital tools must be co-created and adopted, harmonising patient needs and preferences with communication objectives and clinical targets.

### Strengths and limitations of the study

Despite a limited sample size (*n* = 14), the study`s purposive sampling strategy ensured the collection of rich, nuanced, and comprehensive data on remote PCC interventions for older persons living with frailty [32]. The purposive sampling strategy ensured participant diversity across demographic variables (sex, age, living situations, civil status, education level and use of digital tools), thereby enhancing the transferability of the study’s findings to older persons living with mild-to-moderate frailty and similar conditions [53]. In addition, including older adults with visual or hearing impairments demonstrates the accessibility potential of remote PCC interventions. Methodological rigour was ensured through multiple strategies: (1) detailed description of the analytical process, (2) independent data analysis by two researchers and further validating the interpretations by a third researcher, (3) researchers’ reflexivity and (4) comprehensive incorporation of participants’ quotations. The combined approaches served to bolster the study’s trustworthiness [54]. However, the transferability of our findings is constrained by selection bias, as most participants from the main IHOPe study presented with mild-to-moderate frailty. Consequently, the results may not accurately reflect the experiences of older persons living with severe frailty and cognitive impairment. Further research should target the inclusion of these underrepresented populations (e.g., older persons living with cognitive deficits or severe frailty) to validate these findings across the entire frailty spectrum.

Contemporary healthcare systems face the significant challenge of developing integrated technological solutions for care management and delivery that meaningfully engage older adults while addressing their unique needs and preferences [20]. The co-creation of accessible and user-friendly digital devices, the cultivation of trust with older people and the careful consideration of ethical implications within person-centred approaches are crucial elements in eHealth intervention planning [52, 55].

## Conclusions

This study confirms the pivotal role of person-centred conversations in facilitating remote PCC delivery via telephone and digital platforms. A person-centred ethical approach, facilitated by trusting, empathetic and accessible communication, solidified and enhanced available resources, boosting security among older persons living with frailty. However, digital health care communication tools may not suit all older adults. Prioritising patient choice and self-determination within diverse care settings is crucial. Continued development of cost-effective, user-friendly digital solutions is essential to meeting older adults’ varied needs and abilities, particularly those living with frailty.

## Supplementary Information

Supplementary Material 1

## Data Availability

Due to participant confidentiality agreements, the datasets generated and analysed in this study are not publicly accessible. However, anonymised data may be obtained from the corresponding author upon reasonable request. Data are covered by the Public Access to Information and Secrecy Act, and a confidentiality assessment will be performed at each request. Permission from the University of Gothenburg, the Institute of Health and Care Science, must be obtained before data can be accessed. Access can be obtained by contacting the Swedish National Data Service (SND), University of Gothenburg, Box 463, 405 30 Gothenburg, Sweden. Tel. \u0000+ \u000046 31\u00006\u0000-786 10 00. E-mail: snd@gu.se.

## Abbreviations

GPCC: Centre for Person-Centred Care, University of Gothenburg
PCC: Person-centred care
NASSS: Non-adoption, Abandonment, Scale-up, Spread, and Sustainability
IHOPe: Initiating Health Planning with and for Older People through e-Health
HCP: Health Care Professional
FRESH: A screening instrument for frailty in older persons developed by the FRESH (Frail Elderly Support Research Group)
CFS: Clinical Frailty Scale
COVID-19: Coronavirus Disease 2019
