# Supplementary material for: Experiences of a remote, person-centred intervention in older persons living with frailty - a qualitative study
Source: BMC Geriatr. 2025 Oct 15;25:779. doi: 10.1186/s12877-025-06509-0 (PMC12522385; doi:10.1186/s12877-025-06509-0)
Supplement: Supplementary file 1 — Supplementary Material 1 [file 12877_2025_6509_MOESM1_ESM.docx]

**Supplemental material: Interview guide**

*Would you like to tell me about your thoughts and expectations after reading the information sheet about the remote person-centred care intervention?*

Thereafter the interviewee was encouraged to describe his/her experiences of the intervention and its components:

*Please describe a concrete situation, event or moment where this approach proved advantageous or challenging.*

*Can you give an example, a concrete situation/events/ moments, where you experienced this approach as an opportunity and advantage? For example, the phone calls and the interactions and communication via the digital platform?*

*Can you give an example, a concrete situation/events/moments, where you experienced this approach as an obstacle or difficult? For example, the phone calls and the interactions and communication via the digital platform*.

The interview ended with a final question:

*Is there anything more you like to tell me about the study and its components?*

The interviewer used probes/follow-up questions to get as rich data as possible, for example, *tell me more about that, elaborate, what do you mean,* and tried to adapt a flexibility to allow unexpected experiences and stories to emerge.
